# Analyses of more than 60,000 exomes questions the role of numerous genes previously associated with dilated cardiomyopathy

**DOI:** 10.1002/mgg3.245

**Published:** 2016-09-17

**Authors:** Nina Nouhravesh, Gustav Ahlberg, Jonas Ghouse, Charlotte Andreasen, Jesper H. Svendsen, Stig Haunsø, Henning Bundgaard, Peter E. Weeke, Morten S. Olesen

**Affiliations:** ^1^ Laboratory of Molecular CardiologyDepartment of CardiologyThe Heart CentreUniversity Hospital of CopenhagenRigshospitaletCopenhagenDenmark; ^2^ Department of Clinical MedicineFaculty of Medicine and Health SciencesUniversity of CopenhagenCopenhagenDenmark; ^3^ Unit for Inherited Cardiac DiseasesThe Heart CenterNational University HospitalsRigshospitaletCopenhagenDenmark

**Keywords:** Exome, false‐positive variants, familial dilated cardiomyopathy, next‐generation sequencing, NGS

## Abstract

**Background:**

Hundreds of genetic variants have been described as disease causing in dilated cardiomyopathy (DCM). Some of these associations are now being questioned. We aimed to identify the prevalence of previously DCM associated variants in the Exome Aggregation Consortium (ExAC), in order to identify potentially false‐positive DCM variants.

**Methods:**

Variants listed as DCM disease‐causing variants in the Human Gene Mutation Database were extracted from ExAC. Pathogenicity predictions for these variants were mined from dbNSFP v 2.9 database.

**Results:**

Of the 473 DCM variants listed in HGMD, 148 (31%) were found in ExAC. The expected number of individuals with DCM in ExAC is 25 based on the prevalence in the general population. Yet, 35 variants were found in more than 25 individuals. In 13 genes, we identified all variants previously associated with DCM; four genes contained variants above our estimated cut‐off. Prediction tools found ExAC variants to be significantly more tolerated when compared to variants not found in ExAC (*P* = 0.004).

**Conclusion:**

In ExAC, we identified a higher genotype prevalence of variants considered disease‐causing than expected. More importantly, we found 13 genes in which all variants previously associated with DCM were identified in ExAC, questioning the association of these genes with the monogenic form of DCM.

## Introduction

Familial dilated cardiomyopathy (DCM) is a heart muscle disorder defined by the presence of dilatation and systolic impairment of the left or both ventricles, in the absence of hypertension, coronary artery disease, or valvular abnormalities (Codd et al. 1989; Elliott et al. 2008; Jefferies and Towbin 2010). Dilated cardiomyopathy is a known risk factor for sudden cardiac death (SCD), a major cause of heart failure (HF) and end‐stage disease may necessitate cardiac transplantation in both children and adults (Elliott et al. 2008; Everly 2008; Jefferies and Towbin 2010). Dilated cardiomyopathy is a disorder affecting approximately 1:2500 individuals (Codd et al. 1989; Maron et al. 2006). Factors, such as myocarditis, radiation, or toxins may lead to the development of the DCM phenotype. However, a large proportion of cases are idiopathic and a genetic etiology is reported in 48% of these cases (Haas et al. 2015).

Remarkable progress has been made regarding the genetic background of DCM, associating the disease with hundreds of rare variants across many genes (Harakalova et al. 2015; The Human Gene Mutation Database 2015). Due to latest advances in the field of genetic testing, recent reports have found variants previously associated with DCM to be either nonpathogenic or low‐frequency polymorphisms (Andreasen et al. 2013). These findings now question the role of many variants associated with the monogenic form of DCM.

In 2014, the Exome Aggregation Consortium (ExAC) published a browser providing exome data on approximately 61,000 individuals, thereby giving knowledge on low‐frequency polymorphisms (Exome Aggregation Consortium).

We aimed to identify possible false‐positive genetic variants previously associated with DCM, by investigating the prevalence of previously reported DCM associated variants in the ExAC data and comparing the prevalence of these variants with the expected prevalence of DCM in the same population.

## Materials and Methods

In ExAC, next‐generation sequencing of protein coding regions of the genome was performed in 60,706 unrelated individuals from different population groups, ultimately divided in South Asians, Europeans (non‐Finnish), Finnish, Africans, East Asians, Latinos and others. No clinical data are available on the ExAC population. The Human Gene Mutation Database (HGMD) was searched with the term “dilated, cardiomyopathy” and missense‐, stop gained‐ and splice variants associated with DCM were identified.(The Human Gene Mutation Database 2015) Variants found in HGMD were then systematically searched for in ExAC.

Based on the DCM prevalence of 1:2500 (Codd et al. 1989; Maron et al. 2006), the estimated number of individuals with DCM in ExAC is approximately 25. Thus, a given variant with complete penetrance can be present 25 times in ExAC and still theoretically be the monogenic cause of DCM.

PolyPhen‐2‐ and SIFT predictions on all identified missense variants, including those not found in ExAC, were extracted from the database dbNSFP v. 2.9 (Dong et al. 2015). Variants were predicted to be “benign”, “possibly damaging”, or “probably damaging” by PolyPhen‐2 and “damaging” or “tolerated” by SIFT. To give the most accurate prediction, we made a logistic regression (LR) model incorporating 10 prediction scores (SIFT, PolyPhen‐2 HDIV, PolyPhen‐2 HVAR, GERP++, MutationTaster, Mutation Assessor, FATHMM, LRT, SiPhy, PhyloP) and the maximum frequency observed in the 1000 genomes populations. This model classified variants as “tolerated”, “damaging”, or “neutral”.

The pathogenicity of stop gained and splice variants were not predicted by prediction tools.

The American College of Medical Genetics and Genomics (ACMG) recently proposed a new set of criteria for interpretation of variants (Richards et al. 2015). They recommended a five‐tier terminology system using the terms “pathogenic”, “likely pathogenic”, “benign”, “likely benign”, and “uncertain significance”. The term “likely” was defined as a 90% certainty. Necessary information was extracted from ExAC, HGMD, Ensembl, the Exome Variant Server (EVS), and published literature (The Human Gene Mutation Database 2015; Ensembl; Exome Aggregation Consortium; Exome Variant Server). In silico analysis was used as a tool to incorporate all information and classify variants (In silico ACMG program). This method was used to further examine and classify the variants found in the 13 genes questioned.

Differences in distribution of the LR prediction categories between variants identified in ExAC and variants not identified in ExAC were assessed by a chi‐squared test. A two‐sided *P*‐value <0.05 was considered significant.

## Results

We found 473 variants previously associated with DCM in HGMD and 148 (31%) of these were identified in ExAC (Table 1). Eight variants were stop gained variants, nine were splice variants, and 131 were missense variants (Table S1). The missense variants were found in both homozygous and heterozygous individuals. The stop gained variants were all heterozygous and found in five different genes (*BAG3*,* LAMA4*,* PLN*,* TTN*, and *VPS13A*) and the splice variants were found in four different genes (FLNC, *TNNT2*,* TTN*, and *LMNA*), all heterozygous for the variants as well.

**Table 1:** DCM‐associated variants identified in HGMD and found in ExAC

Gene	No. of variants found in ExAC	No. of variants found in HGMD
*ABCC9*	1	1
*ACTN2*	3	4
*ANKRD1*	2	5
*BAG3*	6	15
*CRYAB*	2	3
*CSRP3*	3	3
*DES*	5	8
*DMD*	2	9
*DSP*	2	8
*FHOD3*	1	1
*FLNC*	1	1
*FLT1*	1	1
*ISL1*	1	1
*LAMA2*	1	2
*LAMA4*	2	2
*LDB3*	8	12
*LMNA*	11	80
*MURC*	4	4
*MYBPC3*	13	32
*MYH6*	3	4
*MYH7*	20	71
*MYPN*	5	10
*NCOA6*	1	2
*NEBL*	4	4
*NEXN*	1	2
*PLN*	3	5
*PRDM16*	3	4
*PSEN1*	1	2
*RAF1*	3	6
*RBM20*	3	16
*SCN5A*	3	8
*SGCD*	1	1
*SYNE1*	1	1
*TMPO*	1	1
*TNNI3*	1	8
*TNNT2*	4	20
*TPM1*	1	10
*TTN*	15	55
*TXNRD2*	1	2
*VCL*	3	4
*VPS13A*	1	1

The 148 variants were identified in 7928 alleles corresponding to 7743 individuals carrying a variant when taking homozygous alleles into account. However, this is only valid if we assume that no individual in the ExAC population has more than one DCM‐associated variant. On average, the 148 variants found in ExAC have been screened for in 55,366 individuals. This corresponds to a DCM genotype prevalence of 1:7 (7743:55,366) in ExAC.

We found 35 variants present in 25 individuals or more (Table 2), corresponding to a DCM genotype prevalence of 1:8 (7270: 55,366) (Table 2). When eliminating these variant from the analysis, we find a DCM genotype prevalence of 1: 117 (473:55,366) in ExAC. Furthermore, there were 13 genes in which each HGMD listed variant was identified in ExAC; hereof, four genes (*TMPO*,* NEBL*,* FLT1*, and *ISL1*) contained variants found in more than 25 individuals (Table 3).

**Table 2:** DCM‐associated variants identified in the Exome Aggregation Consortium with an allele count above 25

Gene	Variant	Amino acid	Variant type	Total AC/AN	Total allele frequency	LR prediction	Homoz.
*ACTN2*	c.26A>G	Q9R	Missense	83/117828	0.0007044	Neutral	0
*ANKRD1*	c.319G>T	V107L	Missense	87/120792	0.0007202	Tolerated	0
	c.313C>T	P105S	Missense	28/120528	0.0002323	Tolerated	0
*BAG3*	c.280A>T	I94F	Missense	92/121376	0.000758	Damaging	0
	c.1138C>T	P380S	Missense	180/121378	0.001483	Neutral	2
*CRYAB*	c.460G>A	G154S	Missense	93/121406	0.000766	Tolerated	0
*CSRP3*	c.10T>C	W4R	Missense	287/121088	0.0023702	Tolerated	0
*DES*	c.1375G>A	V459I	Missense	368/121350	0.0030326	Neutral	8
*DMD*	c.9682T>C	F3228L	Missense	59/83652	0.0007053	Tolerated	0
	c.5016T>A	N1672K	Missense	660/87371	0.007554	Tolerated	16
*DSP*	c.6881C>G	A2294G	Missense	103/121412	0.0008484	Damaging	0
*FLT1*	c.162G>C	R54S	Missense	295/121258	0.0024328	Tolerated	3
*ISL1*	c.755A>G	N252S	Missense	60/118562	0.0005061	Damaging	0
*LAMA2*	c.2462C>T	T821M	Missense	246/120992	0.0020332	Neutral	2
*LDB3*	c.566C>T	S189L	Missense	68/119136	0.0005708	Unknown	0
	c.349G>A	D117N	Missense	549/120546	0.0045543	Neutral	1
	c.1051A>G	T351A	Missense	58/113628	0.0005104	Neutral	0
	c.1535A>C	Q512P	Missense	76/120808	0.0006291	Neutral	0
	c.1672A>G	I558V	Missense	46/76462	0.0006016	Neutral	0
*LMNA*	c.1930C>T	R644C	Missense	145/116680	0.0012427	Unknown	1
*MYBPC3*	c.3682C>T	R1228C	Missense	26/120584	0.0002156	Tolerated	0
	c.961G>A	V321M	Missense	37/80000	0.0004625	Tolerated	0
*MYH6*	c.3010G>T	A1004S	Missense	119/121412	0.0009801	Tolerated	0
*MYPN*	c.59A>G	Y20C	Missense	111/121136	0.0009163	Damaging	0
	c.3335C>T	P1112L	Missense	368/121276	0.0030344	Damaging	3
	c.3583G>A	V1195M	Missense	31/120970	0.0002563	Damaging	0
*NCOA6*	c.3526A>G	T1176A	Missense	34/121394	0.0002801	Tolerated	0
*NEBL*	c.1775C>A	A592V	Missense	30/116884	0.0002567	Tolerated	0
	c.604G>A	G202R	Missense	258/121218	0.0021284	Tolerated	0
	c.180G>C	K60N	Missense	467/121112	0.0038559	Tolerated	0
*PRDM16*	c.3301G>A	V1100M	Missense	428/120002	0.0035666	Neutral	7
*RBM20*	c.2662G>A	D888N	Missense	59/20800	0.0028365	Neutral	1
*SCN5A*	c.5507T>C	I1835T	Missense	28/120774	0.0002318	Damaging	0
	c.1336G>A	E446K	Missense	82/113512	0.0007224	Damaging	0
*TMPO*	c.2068C>T	R690C	Missense	1794/119000	0.0150756	Neutral	141

AN, allele number, shows how many individuals were exome sequenced at the given locus; AC, allele count, a count of how many alleles of a given variant was found; LR, logistic regression; Homoz., homozygous.

**Table 3:** Genes in which all variants previously associated with DCM were found in the Exome Aggregation Consortium

Gene	Variant	Amino acid	Variant type	Total AC/AN	Total allele frequency	LR prediction	ACMG prediction	Homoz.
*ABCC9*	c.4537G>A	A1513T	Missense	6/120638	4.9736E‐05	Damaging	Uncertain significance	0
*CSRP3*	c.206A>G	K69R	Missense	2/121324	1.6485E‐05	Damaging	Uncertain significance	0
	c.148G>A	A50T	Missense	5/121014	4.1318E‐05	Damaging	Uncertain significance	0
	c.10T>C	W4R	Missense	287/121088	0.00237018	Tolerated	Benign	0
*FHOD3*	c.3745T>A	Y1249N	Missense	2/120254	1.6631E‐05	Damaging	Uncertain significance	0
*FLNC*	c.3791‐1G>C		Splice variant	1/118498	8.439E‐06		Uncertain significance	0
*FLT1*	c.162G>C	54.R	Missense	295/121258	0.00243283	Tolerated	Benign	3
*ISL1*	c.755A>G	N252S	Missense	60/118562	0.00050606	Damaging	Benign	0
*LAMA4*	c.3217C>T	R1073	Stop‐gain	1/121404	8.237E‐06		Pathogenic	0
	c.2828C>T	P943L	Missense	3/121284	2.4735E‐05	Tolerated	Uncertain significance	0
*MURC*	c.384C>G	N128K	Missense	13/116518	0.00011157	Damaging	Pathogenic	0
	c.418C>T	R140W	Missense	6/120084	4.9965E‐05	Neutral	Uncertain significance	0
	c.458T>C	L153P	Missense	1/121130	8.2556E‐06	Neutral	Uncertain significance	0
	c.1091C>T	S364L	Missense	2/117466	1.7026E‐05	Neutral	Uncertain significance	0
*NEBL*	c.1775C>A	A592V	Missense	30/116884	0.00025666	Tolerated	Likely benign	0
	c.604G>A	G202R	Missense	258/121218	0.0021284	Tolerated	Likely benign	0
	c.383A>G	Q128L	Missense	5/121306	4.1218E‐05	Tolerated	Uncertain significance	0
	c.180G>C	K60N	Missense	467/121112	0.00385594	Tolerated	Uncertain significance	0
*SGCD*	c.212G>C	R71T	Missense	3/114858	2.6119E‐05	Damaging	Uncertain significance	0
*SYNE1*	c.24422G>A	R8141H	Missense	7/111698	6.2669E‐05	Tolerated	Uncertain significance	0
*TMPO*	c.2068C>T	R690C	Missense	1794/119000	0.01507563	Neutral	Likely benign	141
*VPS13A*	c.9403C>T	R3135	Stop‐gain	3/120448	2.4907E‐05		Uncertain significance	0

AN, allele number, shows how many individuals were exome sequenced at the given locus; AC, allele count, a count of how many alleles of a given variant was found; LR, logistic regression; Homoz., homozygous.

The pathogenicity of the variants was determined by Polyphen‐2, SIFT, and LR analysis. Since LR includes SIFT and Polyphen‐2, only LR data are presented here (SIFT and Polyphen‐2 data are presented in Table S2). LR predicted 39 (26%) of the 148 variants to be tolerated, 61 (41%) to be damaging, and 28 (19%) to be neutral. A total of 20 (14%) variants were classified as unknown. Of the remaining 325 DCM‐associated variants 28 (9%) were predicted to be tolerated, 190 (58%) to be damaging, 33 (10%) to be neutral, and 74 (23%) were classified as unknown (Table S1).

When comparing variants found in ExAC with those not found in ExAC, we find a significantly larger number of tolerated variants in ExAC when using LR prediction (*P* = 0.004). However, there was no difference in the number of damaging and neutral variants between the two groups (*P* = 0.07 and *P* = 0.09, respectively).

The pathogenicity of variants found in the 13 genes in which all HGMD listed variants were identified in ExAC was further classified according to the ACMG guidelines. Of the 22 variants, 14 were of uncertain significance, three were classified as likely benign, three as benign, and two as pathogenic (Table 3).

## Discussion

In a recently published exome browser, ExAC, we identified 148 variants previously associated with DCM. This corresponds to 31% of all variants associated with DCM, questioning a substantial proportion of the genetic background. The estimated prevalence of DCM is 1:2500 in the general population, (Codd et al. 1989; Maron et al. 2006) we would therefore expect around 25 individuals to have DCM in the ExAC data. The 148 variants found in ExAC were identified in 7743 individuals, corresponding to a genotype prevalence of 1:7. Thus, the genotype prevalence was 3–400‐fold higher than the expected phenotype prevalence of DCM. This could relate to low penetrance – though unlikely to explain a difference of this size – or it might relate to lower pathogenicity of these variants. We found 35 variants to be present in more than 25 individuals. Recent guidelines stated that an allele frequency which is higher than expected for a disease in a control population is considered a strong indication for a benign interpretation (Richards et al. 2015). Supporting the notion, that these genetic variants are less likely to be monogenetic causes of DCM.

The remaining 113 variants found in ExAC were present in <25 alleles and an interpretation of these variants is therefore more complicated. LR predicted variants found in ExAC to be significantly more tolerated (*P* = 0.004). This could indicate that variants identified in ExAC are less likely disease‐causing in comparison to those not found ExAC.

The most important finding of this study is the identification of 13 genes in which all variants previously associated with DCM were found in ExAC (Table 3). This highly questions the role of these genes in the DCM pathogenesis and further suggests that these genes could be innocent bystanders with regard to DCM. Four genes, *TMPO*,* NEBL*,* FLT1*, and *ISL1*, are specially worth noticing, as variants found in these genes were found in more than 25 individuals. Interestingly, variants in *TMPO* and *FLT1* have previously been identified numerously in a large control population (Andreasen et al. 2013). However, our study validates this in a control population ninefold larger. The four genes, *TMPO*,* NEBL*,* FLT1*, and *ISL1*, are examples of genes initially associated through candidate gene approach due to a plausible association to DCM. *NEBL* and *FLT1* are both implicated in the contraction process of the myocardium, (Zeller et al. 2006; Purevjav et al. 2010) while *ISL1* and *TMPO* are important genes in the prenatal development of the myocardial contractile elements (Taylor et al. 2005; Friedrich et al. 2013). Our study indicates that variants identified in these genes are too frequent to have a causal role in the pathogenicity of DCM.

The new ACMG guidelines provide a more stringent approach with regard to classifying variants. We applied these guidelines on the variants found in the 13 genes questioned and found a large proportion to be of uncertain significance (Table 3), due to either conflicting evidence or lack of evidence (Table S3). This simply demonstrates that we do not have sufficient evidence for classification of these variants.

Due to advances in the genetic field, exome sequencing has become more accessible in recent years. Thus, a large number of reports have been conducted, to separate false‐positive variants from truly disease‐causing variants in different cardiomyopathies and channelopathies (Norton et al. 2012; Refsgaard et al. 2012; Andreasen et al. 2013; Mogensen et al. 2015). All together, these findings suggest a revision of previously DCM‐associated variants and an optimized approach for the clinical application of genetic findings.

Genetic screening is applied in daily clinical practice more than ever, stressing the importance of accurately classifying genetic variants. Presymptomatic testing of relatives for a false‐positive variant will lead to a wrong differentiation between carriers at risk – and non‐carriers not at risk, ultimately leading to devastating outcomes. It is therefore of the outmost importance that variants reported as causative truly are disease‐causing. In addition, our data suggest that a positive genetic test result should not be considered pathogenic beyond doubt but considered a supplement to the clinical assessment.

In conclusion, we found a much higher genotype prevalence of previously DCM‐associated variants than expected in a newly published population‐based exome browser. Our findings suggest that some of these previously DCM‐associated variants could represent false‐positive findings or at best disease modifiers. More importantly, we found 13 genes in which all variants previously associated with DCM were identified, which seriously questions these genes as likely monogenic causes of DCM.

The study was limited by the lack of clinical data. However, the penetrance of the disease and the age at onset is highly variable. Thus, clinical data would not have changed the conclusion of this study (Mestroni et al. 1999). We acknowledge that the cut‐off can be debated. However, the cut‐off is arbitrary and only provided as a tool to highlight variants too numerous, to be a monogenic cause of DCM. We choose a very conservative cut‐off based on the prevalence of DCM (1:2500), as it implicates that all DCM cases in the ExAC browser are caused by one variant, which is indeed unlikely. However, a small overrepresentation of DCM in the ExAC data is possible. Nevertheless, it would be unlikely that this would contribute with more than a few alleles to the total allele count for each reported variant as most DCM variants reported in the literature are very rare variants specific for a family. In other words, we still find 25 as a reasonable cut‐off. The assumption that no individual has more than one DCM‐associated variant in ExAC is of course a limitation. However, this is the condition for the analysis. We rely on the fact that the prevalence of DCM is so high in ExAC, that it is very unlikely that the difference in prevalence of DCM in ExAC and the general population can be explained by individuals carrying more than one variant.

## Conflict of Interest

None declared.

## Supporting information


**Table S1.** DCM‐associated variants identified in the Exome Aggregation Consortium.

**Table S2.** Additional analysis for Polyphen‐2 and SIFT predictions Chi‐squared test for Polyphen‐2 predictions.

**Table S3.** ACMG classification of variants in 13 genes.

## References

[mgg3245-bib-0001] Andreasen, C. , J. B. Nielsen , L. Refsgaard , A. G. Holst , A. H. Christensen , L. Andreasen , et al. 2013 New population‐based exome data are questioning the pathogenicity of previously cardiomyopathy‐associated genetic variants. Eur J Hum Genet 21:918–928. 23299917 10.1038/ejhg.2012.283 PMC3746259

[mgg3245-bib-0002] Codd, M. B. , D. D. Sugrue , B. J. Gersh , and L. J. Melton . 1989 Epidemiology of idiopathic dilated and hypertrophic cardiomyopathy. A population‐based study in Olmsted County, Minnesota, 1975–1984. Circulation 80:564–572. 2766509 10.1161/01.cir.80.3.564

[mgg3245-bib-0003] Dong, C. , P. Wei , X. Jian , R. Gibbs , E. Boerwinkle , K. Wang , et al. 2015 Comparison and integration of deleteriousness prediction methods for nonsynonymous SNVs in whole exome sequencing studies. Hum. Mol. Genet. 24:2125–2137. 25552646 10.1093/hmg/ddu733 PMC4375422

[mgg3245-bib-0004] Elliott, P. , B. Andersson , E. Arbustini , Z. Bilinska , F. Cecchi , P. Charron , et al. 2008 Classification of the cardiomyopathies: a position statement from the European Society Of Cardiology Working Group on Myocardial and Pericardial Diseases. Eur. Heart J. 29:270–276. 17916581 10.1093/eurheartj/ehm342

[mgg3245-bib-0005] Ensembl. Available at: http://www.ensembl.org/index.html.

[mgg3245-bib-0006] Everly, M. J. . 2008 Cardiac transplantation in the United States: an analysis of the UNOS registry. Clin. Transpl. 35–43. 19708444

[mgg3245-bib-0007] Exome Aggregation Consortium (ExAC) , Cambridge, MA (URL: http://exac.broadinstitute.org) accessed March, 2015.

[mgg3245-bib-0008] Exome Variant Server. Available at: http://evs.gs.washington.edu/EVS/

[mgg3245-bib-0009] Friedrich, F. W. , G. Dilanian , P. Khattar , D. Juhr , L. Gueneau , P. Charron , et al. 2013 A novel genetic variant in the transcription factor Islet‐1 exerts gain of function on myocyte enhancer factor 2C promoter activity. Eur. J. Heart Fail. 15:267–276. 23152444 10.1093/eurjhf/hfs178

[mgg3245-bib-0010] Haas, J. , K. S. Frese , B. Peil , W. Kloos , A. Keller , R. Nietsch , et al. 2015 Atlas of the clinical genetics of human dilated cardiomyopathy. Eur. Heart J. 36:1123–1135. 25163546 10.1093/eurheartj/ehu301

[mgg3245-bib-0011] Harakalova, M. , G. Kummeling , A. Sammani , M. Linschoten , A. F. Baas , J. van der Smagt , et al. 2015 A systematic analysis of genetic dilated cardiomyopathy reveals numerous ubiquitously expressed and muscle‐specific genes. Eur. J. Heart Fail. 17:484–493. 25728127 10.1002/ejhf.255

[mgg3245-bib-0012] In silico ACMG program. Available at: http://medschool.umaryland.edu/Genetic_Variant_Interpretation_Tool1.html.

[mgg3245-bib-0013] Jefferies, J. L. , and J. A. Towbin . 2010 Dilated cardiomyopathy. Lancet 375:752–762. 20189027 10.1016/S0140-6736(09)62023-7

[mgg3245-bib-0014] Maron, B. J. , J. A. Towbin , G. Thiene , C. Antzelevitch , D. Corrado , D. Arnett , et al. 2006 Contemporary definitions and classification of the cardiomyopathies: an American Heart Association Scientific Statement from the Council on Clinical Cardiology, Heart Failure and Transplantation Committee; Quality of Care and Outcomes Research and Functional Genomics and Translational Biology Interdisciplinary Working Groups; and Council on Epidemiology and Prevention. Circulation 113:1807–1816. 16567565 10.1161/CIRCULATIONAHA.106.174287

[mgg3245-bib-0015] Mestroni, L. , B. Maisch , W. J. McKenna , K. Schwartz , P. Charron , C. Rocco , et al. 1999 Guidelines for the study of familial dilated cardiomyopathies. Collaborative Research Group of the European Human and Capital Mobility Project on Familial Dilated Cardiomyopathy. Eur. Heart J. 20:93–102. 10099905 10.1053/euhj.1998.1145

[mgg3245-bib-0016] Mogensen, J. , J. P. van Tintelen , S. Fokstuen , P. Elliott , I. M. van Langen , B. Meder , et al. 2015 The current role of next‐generation DNA sequencing in routine care of patients with hereditary cardiovascular conditions: a viewpoint paper of the European Society of Cardiology working group on myocardial and pericardial diseases and members of the European Society of Human Genetics. Eur. Heart J. 36:1367–1370. 25845928 10.1093/eurheartj/ehv122

[mgg3245-bib-0017] Norton, N. , P. D. Robertson , M. J. Rieder , S. Züchner , E. Rampersaud , E. Martin , et al. , and National Heart, Lung and Blood Institute GO Exome Sequencing Project . 2012 Evaluating pathogenicity of rare variants from dilated cardiomyopathy in the exome era. Circ. Cardiovasc. Genet. 5:167–174. 22337857 10.1161/CIRCGENETICS.111.961805 PMC3332064

[mgg3245-bib-0018] Purevjav, E. , J. Varela , M. Morgado , D. L. Kearney , H. Li , M. D. Taylor , et al. 2010 Nebulette mutations are associated with dilated cardiomyopathy and endocardial fibroelastosis. J. Am. Coll. Cardiol. 56:1493–1502. 20951326 10.1016/j.jacc.2010.05.045 PMC2957670

[mgg3245-bib-0019] Refsgaard, L. , A. G. Holst , G. Sadjadieh , S. Haunsø , J. B. Nielsen , and M. S. Olesen . 2012 High prevalence of genetic variants previously associated with LQT syndrome in new exome data. Eur J Hum Genet 20:905–908. 22378279 10.1038/ejhg.2012.23 PMC3400735

[mgg3245-bib-0020] Richards, S. , N. Aziz , S. Bale , D. Bick , S. Das , J. Gastier‐Foster , et al. 2015 Standards and guidelines for the interpretation of sequence variants: a joint consensus recommendation of the American College of Medical Genetics and Genomics and the Association for Molecular Pathology. Genet Med 17:405–424. 25741868 10.1038/gim.2015.30 PMC4544753

[mgg3245-bib-0021] Taylor, M. R. G. , D. Slavov , A. Gajewski , S. Vlcek , L. Ku , P. R. Fain , et al. 2005 Thymopoietin (lamina‐associated polypeptide 2) gene mutation associated with dilated cardiomyopathy. Hum. Mutat. 26:566–574. 16247757 10.1002/humu.20250

[mgg3245-bib-0022] The Human Gene Mutation Database . 2015 Professional 2015.1. Available at: http://www.hgmd.cf.ac.uk/ac/index.php.

[mgg3245-bib-0023] Zeller, R. , B. T. Ivandic , P. Ehlermann , O. Mücke , C. Zugck , A. Remppis , et al. 2006 Large‐scale mutation screening in patients with dilated or hypertrophic cardiomyopathy: a pilot study using DGGE. J Mol Med (Berl) 84:682–691. 16715312 10.1007/s00109-006-0056-2

