# Supplementary material for: Analyses of more than 60,000 exomes questions the role of numerous genes previously associated with dilated cardiomyopathy
Source: Mol Genet Genomic Med. 2016 Sep 17;4(6):617–23. doi: 10.1002/mgg3.245 (PMC5118206; doi:10.1002/mgg3.245)
Supplement: Supplementary file 1 — Table S1. DCM‐associated variants identified in the Exome Aggregation Consortium. [file MGG3-4-617-s001.docx]

Supplementary Table 1 DCM associated variants identified in the Exome Aggregation Consortium

| **Gene** | **Rs number** | **Variant** | **Amino Acid** | **Variant type** | **European (Non-finnish) AC/AN** | **African AC/AN** | **Latino AC/AN** | **East Asian AC/AN** | **Finnish AC/AN** | **South Asian AC/AN** | **Other AC/AN** | **Total AC/AN** | **Total allele frequency** | **Poly-**  **phen-2 prediction** | **Sift Prediction** | **LR prediction** | **Homoz.** |
| --- | --- | --- | --- | --- | --- | --- | --- | --- | --- | --- | --- | --- | --- | --- | --- | --- | --- |
| ABCC9 | rs72559751 | c.4537G>A | A1513T | Missense | 6/66424 | 0/10300 | 0/11450 | 0/8528 | 0/6602 | 0/16434 | 0/900 | 6/120638 | 4.97E-05 | B | T | D | 0 |
| ACTN2 | rs121434525 | c.26A>G | Q9R | Missense | 71/64536 | 0/9854 | 10/11350 | 0/8494 | 2/6392 | 0/16344 | 0/858 | 83/117828 | 0.0007 | B | T | N | 0 |
|  | rs376335356 | c.1046A>T | Q349L | Missense | 0/66720 | 1/10394 | 0/11564 | 0/8654 | 0/6614 | 0/16512 | 0/908 | 1/121366 | 8.24E-06 | B | D | D | 0 |
|  | rs370677725 | c.2323C>T | H775Y | Missense | 3/66738 | 0/10406 | 0/11576 | 0/8654 | 0/6614 | 0/16512 | 0/908 | 3/121408 | 2.47E-05 | B | D | N | 0 |
| ANKRD1 | rs114435632 | c.319G>T | V107L | Missense | 1/66404 | 81/10346 | 3/11536 | 0/8544 | 0/6586 | 1/16470 | 1/906 | 87/120792 | 0.0007 | B | T | T | 0 |
|  | rs148189486 | c.313C>T | P105S | Missense | 25/66252 | 0/10322 | 2/11528 | 0/8500 | 1/6582 | 0/16440 | 0/904 | 28/120528 | 0.0002 | B | T | T | 0 |
| BAG3 | rs387906874 | c.211C>T | R71W | Missense | 9/66712 | 1/10400 | 0/11576 | 1/8654 | 4/6614 | 0/16512 | 0/908 | 15/121376 | 0.0001 | PrD | D | N | 0 |
|  | rs145393807 | c.280A>T | I94F | Missense | 89/66712 | 1/10406 | 0/11574 | 0/8652 | 2/6614 | 0/16512 | 0/906 | 92/121376 | 0.0008 | PrD | D | D | 0 |
|  | null | c.343C>T | P115S | Missense | 1/66618 | 0/10390 | 0/11570 | 0/8648 | 0/6612 | 0/16510 | 0/906 | 1/121254 | 8.25E-06 | B | T | N | 0 |
|  | rs387906875 | c.367C>T | R123* | Stop-gain | 2/66492 | 0/10348 | 0/11570 | 0/8644 | 0/6614 | 0/16510 | 0/906 | 2/121084 | 1.65E-05 |  |  |  | 0 |
|  | rs397514506 | c.652C>T | R218W | Missense | 2/66178 | 0/10260 | 0/11544 | 5/8614 | 1/6612 | 0/16492 | 0/902 | 8/120602 | 6.63E-05 | PrD | T | N | 0 |
|  | rs144692954 | c.1138C>T | P380S | Missense | 6/66716 | 114/10402 | 4/11576 | 1/8652 | 1/6614 | 54/16512 | 0/906 | 180/121378 | 0.0015 | U | T | N | 2 |
| CRYAB | rs141638421 | c.470G>A | R157H | Missense | 1/66736 | 1/10406 | 0/11578 | 9/8654 | 0/6614 | 0/16510 | 0/908 | 11/121406 | 9.06E-05 | PrD | D | D | 0 |
|  | rs150516929 | c.460G>A | G154S | Missense | 85/66736 | 1/10406 | 2/11578 | 0/8654 | 5/6614 | 0/16510 | 0/908 | 93/121406 | 0.0008 | B | T | T | 0 |
| CSRP3 | rs137852764 | c.206A>G | K69R | Missense | 1/66686 | 0/10392 | 0/11572 | 0/8642 | 0/6614 | 1/16510 | 0/908 | 2/121324 | 1.65E-05 | B | D | D | 0 |
|  | rs145300736 | c.148G>A | A50T | Missense | 1/66498 | 1/10326 | 2/11552 | 1/8620 | 0/6614 | 0/16498 | 0/906 | 5/121014 | 4.13E-05 | B | T | D | 0 |
|  | rs45550635 | c.10T>C | W4R | Missense | 258/66602 | 7/10380 | 3/11516 | 0/8628 | 6/6588 | 11/16470 | 2/904 | 287/121088 | 0.0024 | PoD | T | T | 0 |
| DES | rs62636491 | c.893C>T | S298L | Missense | 8/66370 | 1/10344 | 1/11564 | 0/8638 | 0/6612 | 0/16498 | 0/900 | 10/120926 | 8.27E-05 | PrD | T | D | 0 |
|  | rs34337334 | c.934G>A | D312N | Missense | 1/66420 | 20/10342 | 0/11518 | 0/8628 | 0/6548 | 0/16420 | 1/898 | 22/120774 | 0.0002 | PrD | D | D | 0 |
|  | rs62636492 | c.1048C>T | R350W | Missense | 1/66562 | 1/10352 | 0/11566 | 0/8646 | 0/6614 | 0/16512 | 0/908 | 2/121160 | 1.65E-05 | PrD | D | D | 0 |
|  | rs121913002 | c.1353C>G | 451.I | Missense | 6/30906 | 1/5640 | 0/3468 | 0/3920 | 0/2300 | 0/9774 | 0/426 | 7/56434 | 0.0001 | PrD | D | N | 0 |
|  | rs73991549 | c.1375G>A | V459I | Missense | 8/66694 | 323/10398 | 22/11576 | 1/8648 | 0/6614 | 12/16512 | 2/908 | 368/121350 | 0.003 | B | T | N | 8 |
| DMD | rs141392048 | c.9682T>C | F3228L | Missense | 1/45666 | 49/8265 | 8/9095 | 0/6413 | 0/4341 | 1/9272 | 0/600 | 59/83652 | 0.0007 | B | T | T | 0 |
|  | rs16990264 | c.5016T>A | N1672K | Missense | 12/47806 | 598/8501 | 40/9260 | 0/6581 | 0/4511 | 7/10082 | 3/630 | 660/87371 | 0.0076 | PoD | T | T | 16 |
| DSP | rs377715841 | c.5513G>A | R1838H | Missense | 5/66718 | 1/10402 | 1/11576 | 0/8654 | 0/6614 | 0/16504 | 0/908 | 7/121376 | 5.77E-05 | PoD | D | N | 0 |
|  | rs147000526 | c.6881C>G | A2294G | Missense | 65/66740 | 3/10406 | 25/11578 | 0/8654 | 2/6614 | 5/16512 | 3/908 | 103/121412 | 0.0009 | PrD | T | D | 0 |
| FHOD3 | null | c.3745T>A | Y1249N | Missense | 0/66296 | 0/10198 | 0/11440 | 2/8642 | 0/6602 | 0/16180 | 0/896 | 2/120254 | 1.66E-05 | B | T | D | 0 |
| FLNC | null | c.3791-1G>C | . | Splice variant | 1/65266 | 0/9534 | 0/11472 | 0/8534 | 0/6372 | 0/16454 | 0/866 | 1/118498 | 8.44E-06 |  |  |  | 0 |
| FLT1 | rs141440705 | c.162G>C | 54.R | Missense | 156/66656 | 2/10354 | 0/11574 | 0/8650 | 134/6612 | 0/16508 | 3/904 | 295/121258 | 0.0024 | B | T | T | 3 |
| ISL1 | rs121912286 | c.755A>G | N252S | Missense | 41/65560 | 5/9512 | 11/11322 | 0/8520 | 0/6414 | 1/16352 | 2/882 | 60/118562 | 0.0005 | B | T | D | 0 |
| LAMA2 | rs117422805 | c.2462C>T | T821M | Missense | 184/66630 | 5/10392 | 4/11394 | 6/8642 | 24/6586 | 21/16444 | 2/904 | 246/120992 | 0.002 | PrD | D | N | 2 |
| LAMA4 | rs372615994 | c.3217C>T | R1073* | Stop-gain | 1/66740 | 0/10406 | 0/11574 | 0/8652 | 0/6614 | 0/16510 | 0/908 | 1/121404 | 8.24E-06 |  |  |  | 0 |
|  | rs387907365 | c.2828C>T | P943L | Missense | 3/66688 | 0/10392 | 0/11562 | 0/8626 | 0/6606 | 0/16506 | 0/904 | 3/121284 | 2.47E-05 | PoD | T | T | 0 |
| LDB3 | rs121908338 | c.356C>T | A119V | Missense | 1/63308 | 0/9450 | 0/11422 | 0/8354 | 0/6566 | 0/16388 | 0/868 | 1/116356 | 8.59E-06 | B | T | U | 0 |
|  | rs397517223 | c.566C>T | S189L | Missense | 47/65318 | 0/9814 | 7/11544 | 0/8584 | 1/6604 | 12/16382 | 1/890 | 68/119136 | 0.000571 | B | T | U | 0 |
|  | rs45487699 | c.349G>A | D117N | Missense | 286/66564 | 151/9778 | 54/11568 | 0/8616 | 0/6614 | 51/16510 | 7/896 | 549/120546 | 0.004554 | B | T | N | 1 |
|  | rs200796750 | c.1049C>T | T350I | Missense | 14/62290 | 0/9558 | 0/11204 | 0/8442 | 0/5774 | 0/16306 | 0/836 | 14/114410 | 0.000122 | U | T | U | 0 |
|  | rs138251566 | c.1051A>G | T351A | Missense | 35/61912 | 0/9480 | 19/11096 | 0/8374 | 0/5670 | 4/16266 | 0/830 | 58/113628 | 0.00051 | U | T | N | 0 |
|  | rs138951890 | c.1535A>C | Q512P | Missense | 44/66262 | 1/10332 | 2/11566 | 0/8636 | 23/6604 | 4/16512 | 2/896 | 76/120808 | 0.000629 | B | T | N | 0 |
|  | rs372331627 | c.1672A>G | I558V | Missense | 5/41660 | 0/6664 | 1/6264 | 0/5148 | 0/3412 | 40/12734 | 0/580 | 46/76462 | 0.000602 | PrD | D | N | 0 |
|  | rs45514002 | c.2017G>A | D673N | Missense | 6/66730 | 1/10404 | 0/11562 | 14/8654 | 0/6614 | 0/16486 | 0/908 | 21/121358 | 0.000173 | PrD | T | N | 0 |
| LMNA | rs267607626 | c.565C>T | R189W | Missense | 0/66642 | 0/10372 | 1/11554 | 1/8652 | 0/6592 | 0/16506 | 0/906 | 2/121224 | 1.65E-05 | B | T | D | 0 |
|  | rs57045855 | c.575A>G | D192G | Missense | 3/66602 | 0/10374 | 0/11546 | 0/8650 | 2/6408 | 0/16510 | 0/896 | 5/120986 | 4.13E-05 | PoD | D | D | 0 |
|  | rs80356807 | null | . | Splice variant | 0/66624 | 0/9878 | 0/11576 | 0/8652 | 0/6614 | 1/16512 | 0/902 | 1/120758 | 8.28E-06 |  |  |  | 0 |
|  | null | c.992G>A | R331Q | Missense | 1/64850 | 0/9664 | 0/11488 | 0/8584 | 0/6406 | 1/16488 | 0/878 | 2/118358 | 1.69E-05 | PoD | T | D | 0 |
|  | rs370656306 | c.1001G>A | S334N | Missense | 1/65122 | 0/9726 | 0/11508 | 0/8602 | 0/6448 | 0/16498 | 0/884 | 1/118788 | 8.42E-06 | B | T | D | 0 |
|  | rs267607576 | c.1163G>A | R388H | Missense | 1/65546 | 0/9982 | 0/11498 | 0/8548 | 0/6492 | 0/16502 | 0/888 | 1/119456 | 8.37E-06 | B | D | D | 0 |
|  | rs150840924 | c.1303C>T | R435C | Missense | 1/63644 | 0/10040 | 0/11042 | 0/8274 | 0/5902 | 0/16160 | 0/836 | 1/115898 | 8.63E-06 | PoD | D | D | 0 |
|  | rs201583907 | c.1567G>A | G523R | Missense | 7/38488 | 1/6832 | 0/5380 | 0/4856 | 0/3018 | 0/12102 | 0/562 | 8/71238 | 0.000112 | PrD | D | D | 0 |
|  | rs60890628 | c.1711C>A | R571S | Missense | 0/6444 | 0/2072 | 0/348 | 1/652 | 0/30 | 0/7894 | 0/166 | 1/17606 | 5.68E-05 | U | T | N | 0 |
|  | rs142000963 | c.1718C>T | S573L | Missense | 9/57438 | 0/7222 | 1/10654 | 0/8074 | 0/5008 | 1/15096 | 0/740 | 11/104232 | 0.000106 | B | D | D | 0 |
|  | rs80338938 | c.1930C>T | R644C | Missense | 106/63724 | 7/9554 | 4/11402 | 0/8540 | 1/6188 | 27/16410 | 0/862 | 145/116680 | 0.001243 | PoD | D | U | 1 |
| MURC | rs143268013 | c.384C>G | N128K | Missense | 0/63574 | 13/9246 | 0/11394 | 0/8510 | 0/6600 | 0/16320 | 0/874 | 13/116518 | 0.000112 | PrD | D | D | 0 |
|  | null | c.418C>T | R140W | Missense | 6/65942 | 0/10272 | 0/11454 | 0/8588 | 0/6572 | 0/16358 | 0/898 | 6/120084 | 5E-05 | PoD | D | N | 0 |
|  | rs370997194 | c.458T>C | L153P | Missense | 0/66578 | 1/10368 | 0/11544 | 0/8638 | 0/6602 | 0/16498 | 0/902 | 1/121130 | 8.26E-06 | B | T | N | 0 |
|  | null | c.1091C>T | S364L | Missense | 1/64590 | 1/9434 | 0/11404 | 0/8340 | 0/6554 | 0/16270 | 0/874 | 2/117466 | 1.7E-05 | B | D | Nl | 0 |
| MYBPC3 | rs201312636 | c.3682C>T | R1228C | Missense | 10/66622 | 13/9766 | 2/11568 | 1/8612 | 0/6614 | 0/16506 | 0/896 | 26/120584 | 0.000216 | PrD | D | T | 0 |
|  | null | c.2909G>A | R970Q | Missense | 1/15178 | 0/2842 | 0/1302 | 0/1674 | 0/1106 | 0/8384 | 0/274 | 1/30760 | 3.25E-05 | PoD | T | T | 0 |
|  | null | c.1985T>C | V662A | Missense | 1/66562 | 0/9760 | 0/11526 | 0/8616 | 0/6606 | 0/16436 | 0/896 | 1/120402 | 8.31E-06 | PrD | D | T | 0 |
|  | rs397515939 | c.1960C>T | R654C | Missense | 1/66648 | 0/9798 | 0/11560 | 0/8624 | 0/6612 | 0/16444 | 0/898 | 1/120584 | 8.29E-06 | B | D | T | 0 |
|  | null | c.1309G>A | V437L | Missense | 1/61376 | 0/8540 | 0/10208 | 0/8028 | 0/5864 | 0/14934 | 0/804 | 1/109754 | 9.11E-06 | B | T | T | 0 |
|  | null | c.1153G>A | V385M | Missense | 0/56022 | 1/7528 | 0/9158 | 0/7302 | 0/4692 | 0/13470 | 0/708 | 1/98880 | 1.01E-05 | PrD | D | T | 0 |
|  | rs397516086 | c.994G>A | E332K | Missense | 0/57456 | 0/8364 | 1/9316 | 0/7474 | 0/5480 | 0/14236 | 0/752 | 1/103078 | 9.7E-06 | PoD | D | T | 0 |
|  | rs200119454 | c.961G>A | V321M | Missense | 36/45024 | 1/6832 | 0/6552 | 0/5714 | 0/4320 | 0/10974 | 0/584 | 37/80000 | 0.000463 | B | D | T | 0 |
|  | rs397516075 | c.814C>T | R272C | Missense | 2/11516 | 0/2316 | 0/654 | 0/980 | 0/156 | 0/8038 | 0/210 | 2/23870 | 8.38E-05 | PrD | D | T | 0 |
|  | null | c.713G>A | R238H | Missense | 0/59988 | 0/8584 | 5/10018 | 3/7896 | 0/5686 | 0/14698 | 0/794 | 8/107664 | 7.43E-05 | PrD | D | D | 0 |
|  | rs397516050 | c.442G>A | G148R | Missense | 1/10814 | 0/2372 | 0/664 | 0/900 | 0/832 | 0/8010 | 0/214 | 1/23806 | 4.2E-05 | B | T | T | 0 |
|  | rs569824900 | c.251G>A | G84D | Missense | 0/13106 | 0/2720 | 0/854 | 0/1058 | 0/966 | 1/7174 | 0/220 | 1/26098 | 3.83E-05 | PrD | D | T | 0 |
|  | null | c.121C>T | R41C | Missense | 2/48422 | 0/6394 | 1/8094 | 0/6230 | 0/3470 | 0/12672 | 0/608 | 3/85890 | 3.49E-05 | PoD | D | T | 0 |
| MYH6 | rs267606905 | c.4369G>A | E1457K | Missense | 1/66104 | 0/10138 | 1/11498 | 0/8602 | 0/6602 | 0/16420 | 0/898 | 2/120262 | 1.66E-05 | PoD | D | D | 0 |
|  | rs137983703 | c.4318G>C | A1440S | Missense | 1/66726 | 0/10406 | 0/11574 | 0/8654 | 0/6614 | 0/16512 | 0/908 | 1/121394 | 8.24E-06 | B | D | D | 0 |
|  | rs143978652 | c.3010G>T | A1004S | Missense | 79/66740 | 3/10406 | 31/11578 | 0/8654 | 4/6614 | 1/16512 | 1/908 | 119/121412 | 0.00098 | B | D | T | 0 |
| MYH7 | null | c.5704G>A | E1902K | Missense | 1/66740 | 0/10406 | 0/11578 | 1/8654 | 0/6614 | 0/16512 | 0/908 | 2/121412 | 1.65E-05 | PoD | D | D | 0 |
|  | rs201865159 | c.5494C>T | R1832C | Missense | 5/66692 | 0/10388 | 1/11572 | 0/8642 | 0/6608 | 0/16512 | 0/906 | 6/121320 | 4.95E-05 | PrD | D | D | 0 |
|  | null | c.5030G>A | R1677H | Missense | 6/66726 | 0/10402 | 0/11572 | 0/8654 | 0/6612 | 0/16512 | 0/906 | 6/121384 | 4.94E-05 | PrD | D | D | 0 |
|  | rs370328209 | c.4985G>A | R1662H | Missense | 4/66716 | 1/10396 | 1/11562 | 1/8652 | 0/6598 | 0/16510 | 0/906 | 7/121340 | 5.77E-05 | B | T | T | 0 |
|  | rs397516232 | c.4900C>T | R1634C | Missense | 1/66738 | 6/10402 | 1/11574 | 0/8654 | 0/6614 | 1/16512 | 0/906 | 9/121400 | 7.41E-05 | PrD | D | D | 0 |
|  | null | c.4606G>A | E1536K | Missense | 0/66650 | 0/10358 | 0/11564 | 0/8652 | 0/6614 | 1/16494 | 0/904 | 1/121236 | 8.25E-06 | PoD | D | D | 0 |
|  | rs397516211 | c.4348G>A | D1450N | Missense | 1/66696 | 0/10382 | 0/11568 | 0/8652 | 0/6614 | 0/16510 | 0/908 | 1/121330 | 8.24E-06 | PoD | D | D | 0 |
|  | null | c.4030C>T | R1344W | Missense | 1/66144 | 0/10254 | 0/11552 | 0/8624 | 0/6588 | 1/16492 | 0/902 | 2/120556 | 1.66E-05 | B | D | D | 0 |
|  | rs397516198 | c.3994G>A | A1332T | Missense | 2/64922 | 0/9858 | 0/11456 | 0/8520 | 0/6388 | 0/15718 | 0/874 | 2/117736 | 1.7E-05 | PoD | D | D | 0 |
|  | rs397516196 | c.3856G>A | E1286K | Missense | 2/66740 | 0/10406 | 0/11576 | 0/8654 | 0/6614 | 0/16512 | 0/906 | 2/121408 | 1.65E-05 | PrD | D | D | 0 |
|  | rs397516189 | c.3626A>G | N1209S | Missense | 0/65888 | 0/10244 | 0/11458 | 0/8526 | 0/6534 | 1/16462 | 0/900 | 1/120012 | 8.33E-06 | PoD | T | D | 0 |
|  | rs397516187 | c.3578G>A | R1193H | Missense | 1/51248 | 0/7544 | 0/8702 | 0/6502 | 0/4990 | 0/15080 | 0/718 | 1/94784 | 1.06E-05 | PrD | D | D | 0 |
|  | null | c.3158G>A | R1053Q | Missense | 0/66740 | 0/10404 | 0/11576 | 0/8652 | 9/6604 | 0/16512 | 0/908 | 9/121396 | 7.41E-05 | PrD | D | D | 0 |
|  | null | c.3152C>T | A1051V | Missense | 1/66740 | 0/10404 | 0/11574 | 0/8654 | 0/6606 | 1/16512 | 0/908 | 2/121398 | 1.65E-05 | B | T | D | 0 |
|  | null | c.3056C>A | T1019N | Missense | 0/66740 | 0/10406 | 1/11578 | 0/8654 | 0/6614 | 0/16512 | 0/908 | 1/121412 | 8.24E-06 | PoD | T | T | 0 |
|  | null | c.2710C>T | R904C | Missense | 1/66730 | 0/10406 | 0/11572 | 0/8654 | 0/6612 | 0/16512 | 0/906 | 1/121392 | 8.24E-06 | PrD | D | D | 0 |
|  | null | c.1772T>C | I591T | Missense | 1/66738 | 0/10406 | 0/11574 | 0/8654 | 0/6614 | 0/16512 | 0/908 | 1/121406 | 8.24E-06 | B | T | T | 0 |
|  | null | c.1325G>A | R442H | Missense | 2/66738 | 0/10406 | 0/11578 | 0/8652 | 0/6614 | 0/16510 | 0/908 | 2/121406 | 1.65E-05 | B | D | D | 0 |
|  | null | c.1129G>A | G377S | Missense | 0/66728 | 0/10404 | 0/11578 | 1/8648 | 0/6612 | 2/16512 | 0/908 | 3/121390 | 2.47E-05 | PoD | D | D | 0 |
|  | rs397516261 | c.706G>A | V236I | Missense | 1/66740 | 0/10406 | 0/11578 | 0/8654 | 0/6614 | 0/16512 | 0/908 | 1/121412 | 8.24E-06 | B | T | T | 0 |
| MYPN | rs140148105 | c.59A>G | Y20C | Missense | 94/66608 | 4/10360 | 11/11570 | 0/8644 | 0/6612 | 1/16434 | 1/908 | 111/121136 | 0.000916 | PrD | U | D | 0 |
|  | rs199476411 | c.2644G>A | A882T | Missense | 0/66732 | 1/10406 | 0/11570 | 0/8624 | 0/6614 | 0/16512 | 0/908 | 1/121366 | 8.24E-06 | B | U | N | 0 |
|  | rs71584501 | c.3263G>A | R1088H | Missense | 0/66658 | 0/10368 | 1/11558 | 0/8630 | 0/6612 | 0/16510 | 0/906 | 1/121242 | 8.25E-06 | PoD | U | N | 0 |
|  | rs71534278 | c.3335C>T | P1112L | Missense | 217/66696 | 15/10404 | 30/11542 | 2/8624 | 3/6598 | 93/16504 | 8/908 | 368/121276 | 0.003034 | PrD | U | D | 3 |
|  | rs71534280 | c.3583G>A | V1195M | Missense | 1/66488 | 15/10300 | 1/11548 | 0/8632 | 0/6612 | 14/16488 | 0/902 | 31/120970 | 0.000256 | PrD | U | D | 0 |
| NCOA6 | rs186687743 | c.3526A>G | T1176A | Missense | 1/66740 | 10406 | 0/11568 | 27/8652 | 0/6614 | 5/16506 | 0/908 | 34/121394 | 0.00028 | B | T | T | 0 |
| NEBL | rs146275785 | c.1775C>A | A592V | Missense | 2/64254 | 18/10104 | 0/11004 | 0/8316 | 0/6418 | 0/15910 | 0/878 | 30/116884 | 0.000257 | B | T | T | 0 |
|  | rs137973321 | c.604G>A | G202R | Missense | 208/66660 | 9/10386 | 21/11506 | 0/8646 | 14/6604 | 3/16508 | 3/908 | 258/121218 | 0.002128 | B | T | T | 0 |
|  | null | c.383A>G | Q128L | Missense | 5/66696 | 0/10400 | 0/11546 | 0/8646 | 0/6602 | 0/16510 | 0/906 | 5/121306 | 4.12E-05 | PoD | T | T | 0 |
|  | rs41277374 | c.180G>C | K60N | Missense | 423/66580 | 8/10364 | 17/11532 | 0/8632 | 4/6602 | 1316498 | 2/904 | 467/121112 | 0.003856 | PrD | D | T | 0 |
| NEXN | rs137853197 | c.1955A>G | Y652C | Missense | 11/66220 | 0/9670 | 0/11498 | 0/8564 | 0/6608 | 1/16420 | 0/896 | 12/119876 | 0.0001 | PrD | D | D | 0 |
| PLN | null | c.26G>A | R9H | Missense | 1/66342 | 0/10278 | 0/11504 | 0/8596 | 0/6608 | 0/16458 | 0/898 | 1/120684 | 8.29E-06 | PoD | D | D | 0 |
|  | rs397516786 | c.61C>A | P21T | Missense | 3/66600 | 0/10352 | 3/11556 | 1/8626 | 0/6612 | 0/16492 | 0/900 | 7/121138 | 5.78E-05 | PoD | T | N | 0 |
|  | rs111033560 | c.116T>G | L39* | Stop-gain | 1/66694 | 0/10396 | 0/11576 | 0/8648 | 0/6614 | 0/16508 | 0/906 | 1/121342 | 8.24E-06 |  |  |  | 0 |
| PRDM16 | rs200052869 | c.811G>A | E271K | Missense | 4/65144 | 1/9596 | 0/11520 | 0/8544 | 0/6590 | 0/16500 | 0/878 | 5/118772 | 4.21E-05 | B | D | N | 0 |
|  | rs397514744 | c.872C>T | P291L | Missense | 3/64536 | 0/9504 | 0/11482 | 0/8522 | 0/6404 | 0/16410 | 0/866 | 3/117724 | 2.55E-05 | PoD | D | U | 0 |
|  | rs201654872 | c.3301G>A | V1100M | Missense | 2/66270 | 1/9706 | 1/11526 | 1/8590 | 0/6554 | 422/16468 | 1/888 | 428/120002 | 0.003567 | B | T | N | 7 |
| PSEN1 | rs121917809 | c.998A>G | D333G | Missense | 1/66740 | 13/10404 | 0/11578 | 0/8654 | 0/6614 | 0/16512 | 0/908 | 14/121410 | 0.000115 | PoD | T | D | 0 |
| RAF1 | null | c.1922C>T | T641M | Missense | 1/66738 | 0/10406 | 0/11578 | 0/8654 | 0/6614 | 0/16512 | 0/908 | 1/121410 | 8.24E-06 | B | D | T | 0 |
|  | null | c.928A>G | T310A | Missense | 1/66624 | 0/10394 | 1/11570 | 0/8654 | 0/6612 | 0/16512 | 0/908 | 2/121274 | 1.65E-05 | B | T | T | 0 |
|  | null | c.709G>A | A237T | Missense | 2/66268 | 0/10364 | 0/11346 | 0/8568 | 0/6544 | 0/16334 | 0/898 | 2/120322 | 1.66E-05 | B | T | T | 0 |
| RBM20 | rs375798246 | c.2147G>A | R716Q | Missense | 2/8492 | 0/2136 | 0/398 | 0/610 | 0/238 | 4/7910 | 0/194 | 6/19978 | 0.0003 | B | T | N | 0 |
|  | rs201370621 | c.2662G>A | D888N | Missense | 23/8524 | 2/2138 | 0/404 | 0/622 | 32/1592 | 0/7286 | 2/234 | 59/20800 | 0.002837 | PoD | T | N | 1 |
|  | null | c.3616G>A | E1206K | Missense | 0/8422 | 0/2146 | 0/410 | 0/622 | 0/34 | 1/7768 | 0/190 | 1/19592 | 5.1E-05 | B | D | U | 0 |
| SCN5A | rs45563942 | c.5507T>C | I1835T | Missense | 0/66738 | 27/9806 | 0/11576 | 0/8630 | 0/6614 | 1/16512 | 0/898 | 28/120774 | 0.000232 | PoD | D | D | 0 |
|  | rs199473341 | c.3835G>A | V1278I | Missense | 17/66628 | 1/10294 | 0/11574 | 0/8650 | 0/6614 | 2/16404 | 0/898 | 20/121062 | 0.000165 | PrD | D | D | 0 |
|  | rs199473339 | c.1336G>A | E446K | Missense | 21/64886 | 0/9684 | 2/11480 | 0/8550 | 0/6606 | 57/11478 | 2/828 | 82/113512 | 0.000722 | PoD | D | D | 0 |
| SGCD | null | c.212G>C | R71T | Missense | 0/63972 | 0/9330 | 0/10862 | 0/8106 | 3/6504 | 0/15228 | 0/856 | 3/114858 | 2.61E-05 | PrD | T | D | 0 |
| SYNE1 | null | c.24422G>A | R8141H | Missense | 5/60846 | 1/8824 | 1/11288 | 0/8362 | 0/5166 | 0/16438 | 0/774 | 7/111698 | 6.27E-05 | PoD | D | T | 0 |
| TMPO | rs17028450 | c.2068C>T | R690C | Missense | 50/65566 | 17/9598 | 1656/11460 | 50/8494 | 16/6588 | 4/16394 | 1/900 | 1794/119000 | 0.015076 | B | D | N | 141 |
| TNNI3 | null | c.347C>G | A116G | Missense | 1/66386 | 0/9752 | 0/11480 | 0/8616 | 0/6422 | 1/16382 | 0/890 | 2/119928 | 1.67E-05 | B | T | D | 0 |
| TNNT2 | rs483352832 | c.430C>T | R144W | Missense | 3/63750 | 0/9576 | 0/11104 | 0/8502 | 0/5764 | 1/15410 | 0/820 | 4/114926 | 3.48E-05 | PrD | D | D | 0 |
|  | rs74315380 | c.391C>T | R131W | Missense | 1/64180 | 0/9668 | 0/11160 | 0/8508 | 0/5898 | 0/15490 | 0/824 | 1/115728 | 8.64E-06 | PrD | D | D | 0 |
|  | null | null | . | Splice variant | 3/66740 | 0/10406 | 1/11578 | 0/8654 | 0/6614 | 1/16512 | 0/908 | 5/121412 | 4.12E-05 |  |  |  | 0 |
|  | rs397516450 | c.218A>G | N73S | Missense | 2/64864 | 0/9914 | 0/11166 | 0/8604 | 0/6056 | 0/15676 | 0/858 | 2/117138 | 1.71E-05 | PoD | T | D | 0 |
| TPM1 | null | c.250G>A | D84N | Missense | 1/66738 | 0/10406 | 0/11578 | 0/8652 | 0/6614 | 0/16512 | 0/908 | 1/121408 | 8.24E-06 | PoD | D | D | 0 |
| TTN | rs199642423 | c.96206G>A | R32069Q | Missense | 0/66676 | 2/9802 | 0/11532 | 0/8602 | 0/6602 | 1/16510 | 0/900 | 3/120624 | 2.49E-05 | B | D | T | 0 |
|  | rs397517735 | null | . | Splice variant | 1/64584 | 0/9578 | 0/11492 | 0/8536 | 0/6608 | 0/10592 | 0/806 | 1/112196 | 8.91E-06 |  |  |  | 0 |
|  | rs374140736 | c.40579C>T | R13527* | Stop-gain | 0/66336 | 1/9644 | 0/11462 | 0/8466 | 0/6604 | 0/16462 | 0/888 | 1/119862 | 8.34E-06 |  |  |  | 0 |
|  | rs370109572 | c.14563C>T | L4855F | Missense | 4/65692 | 0/9598 | 0/11296 | 0/8388 | 0/6580 | 0/15860 | 0/882 | 4/118296 | 3.38E-05 | U | D | T | 0 |
|  | null | c.10409G>A | G3470D | Missense | 2/65572 | 0/9610 | 0/11262 | 5/8338 | 0/6506 | 0/15948 | 0/876 | 7/118112 | 5.93E-05 | U | D | D | 0 |
|  | rs139517732 | c.160G>A | V54M | Missense | 0/66708 | 0/10402 | 0/11556 | 6/8594 | 0/6614 | 0/16506 | 0/906 | 6/121286 | 4.95E-05 | B | D | T | 0 |
|  | rs397517735 | null | . | Splice variant | 1/64584 | 0/9578 | 0/11492 | 0/8536 | 0/6608 | 0/10592 | 0/806 | 1/112196 | 8.91E-06 |  |  |  | 0 |
|  | null | null | . | Splice variant | 1/63442 | 0/9528 | 0/11160 | 0/7832 | 0/6496 | 0/13202 | 0/836 | 1/112496 | 8.89E-06 |  |  |  | 0 |
|  | rs574660186 | c.62572C>T | R19931* | Stop gained | 1/66650 | 0/9796 | 0/11520 | 0/8490 | 1/6608 | 0/16508 | 0/900 | 2/120472 | 1.66E-05 |  |  |  | 0 |
|  | null | null | . | Splice variant | 1/66476 | 0/9754 | 0/11516 | 0/8472 | 0/6610 | 0/16502 | 0/900 | 1/120230 | 8.32E-06 |  |  |  | 0 |
|  | null | c.58678C-T | R18633* | Stop-gain | 0/66274 | 0/9744 | 0/11416 | 0/8328 | 0/6562 | 1/16374 | 0/884 | 1/119582 | 8.36E-06 |  |  |  | 0 |
|  | rs72646846 | c.56953C>T | R18058* | Stop-gain | 7/66670 | 0/9802 | 1/11498 | 0/8476 | 0/6606 | 0/16510 | 0/900 | 8/120462 | 6.64E-05 |  |  |  | 0 |
|  | rs368219776 | null | . | Splice variant | 4/15632 | 0/3628 | 0/1238 | 0/1960 | 0/2200 | 0/8488 | 0/316 | 4/33462 | 0.00012 |  |  |  | 0 |
|  | null | c.12745C>T | . | Splice variant | 1/66666 | 0/9794 | 0/11532 | 0/8564 | 0/6604 | 0/16508 | 0/898 | 1/120566 | 8.29E-06 |  |  |  | 0 |
|  | rs147879266 | c.13250G>A | S4417N | Missense | 0/66410 | 0/9740 | 0/11528 | 16/8532 | 0/6592 | 0/16426 | 0/892 | 16/120120 | 0.000133 | U | D | T | 0 |
| TXNRD2 | null | c.175G>A | A59T | Missense | 1/12210 | 0/2636 | 0/722 | 0/786 | 0/274 | 0/8048 | 0/218 | 1/24894 | 4.02E-05 | PrD | D | T | 0 |
| VCL | rs373010557 | c.2444A>G | K815R | Missense | 2/66688 | 0/10404 | 0/11572 | 0/8648 | 0/6614 | 0/16510 | 0/908 | 2/121344 | 1.65E-05 | PoD | T | D | 0 |
|  | rs121917776 | c.2923>-T | R975W | Missense | 12/66708 | 0/10402 | 0/11574 | 0/8650 | 2/6612 | 2/16512 | 0/906 | 16/121364 | 0.000132 | PoD | D | N | 0 |
|  | rs373317423 | c.3373C>T | R1125C | Missense | 1/66672 | 2/10378 | 0/11562 | 0/8650 | 0/6614 | 0/16508 | 0/906 | 3/121290 | 2.47E-05 | PoD | D | D | 0 |
| VPS13A | rs148173878 | c.9403C>T | R3135* | Stop-gain | 2/66402 | 1/9876 | 0/11538 | 0/8624 | 0/6612 | 0/16490 | 0/906 | 3/120448 | 2.49E-05 |  |  |  | 0 |

Abbreviations: B: Benign T: Tolerated PrD: Probably damaging PoD: Possibly damaging D: Damaging N: Neutral U: Unknown
