# Supplementary material for: Analyses of more than 60,000 exomes questions the role of numerous genes previously associated with dilated cardiomyopathy
Source: Mol Genet Genomic Med. 2016 Sep 17;4(6):617–23. doi: 10.1002/mgg3.245 (PMC5118206; doi:10.1002/mgg3.245)
Supplement: Supplementary file 2 — Table S2. Additional analysis for Polyphen‐2 and SIFT predictions Chi‐squared test for Polyphen‐2 predictions. [file MGG3-4-617-s002.docx]

Supplementary Table 2 Additional analysis for Polyphen-2 and SIFT predictions

Chi-squared test for Polyphen-2 predictions

|  | Variants found in ExAC | Variants *not* found in ExAC | P-value |
| --- | --- | --- | --- |
| Benign | 53 (36%) | 53 (16%) | p<0.001 |
| Possibly damaging | 35 (24%) | 40 (12%) | p<0.001 |
| Probably damaging | 37 (15%) | 123 (38%) | p<0.001 |

Chi-squared test for SIFT predictions

|  | Variants found in ExAC | Variants *not* found in ExAC | P-value |
| --- | --- | --- | --- |
| Tolerated | 58 (39%) | 76 (23%) | p=0.042 |
| Damaging | 76 (51%) | 201 (62%) | p=0.3 |

.
