# Supplementary material for: Analyses of more than 60,000 exomes questions the role of numerous genes previously associated with dilated cardiomyopathy
Source: Mol Genet Genomic Med. 2016 Sep 17;4(6):617–23. doi: 10.1002/mgg3.245 (PMC5118206; doi:10.1002/mgg3.245)
Supplement: Supplementary file 3 — Table S3. ACMG classification of variants in 13 genes. [file MGG3-4-617-s003.docx]

Supplementary table 3 ACMG classification of variants in 13 genes

| **Gene** | **Variant** | **Effect** | **Evidence** |
| --- | --- | --- | --- |
| *ABCC9* | c.4537G>A | Uncertain evidence - not enough evidence | PS3, BP4 |
| *CSRP3* | c.206A>G | Uncertain evidence - not enough evidence | PS3, PP3, BS4 |
| *CSRP3* | c.148G>A | Uncertain evidence - not enough evidence | PP3 |
| *CSRP3* | c.10T>C | Benign (II) | BP4, BS1, PS3, PP5, BS4 |
| *FHOD3* | c.3745T>A | Uncertain evidence - not enough evidence | PP3, PS3 |
| *FLNC* | null | Uncertain evidence - not enough evidence | BP4, PS3 |
| *FLT1* | c.162G>C | Benign (II) | PS3, BS4, BS1, BP4 |
| *ISL1* | c.755A>G | Benign (II) | BS4, BS1, PP3, PS3 |
| *LAMA4* | c.3217C>T | Pathogenic (Ia) | PVS1, PS3, BS4 |
| *LAMA4* | c.2828C>T | Uncertain evidence - not enough evidence | PS3, BP4 |
| *MURC* | c.384C>G | Pathogenic (II) | PP3, PS3, PP1-S |
| *MURC* | c.418C>T | Uncertain evidence - not enough evidence | PS3 |
| *MURC* | c.458T>C | Uncertain evidence - not enough evidence | PS3, BS4 |
| *MURC* | c.1091C>T | Uncertain evidence - not enough evidence | PS3 |
| *NEBL* | c.1775C>A | Likely benign (I) | BP4, BS1, PS3 |
| *NEBL* | c.604G>A | Likely benign (I) | PS3, BS1, BP4 |
| *NEBL* | c.383A>G | Uncertain evidence - conflicting evidence | BP4, BS1, PS3, PM6 |
| *NEBL* | c.180G>C | Uncertain evidence - conflicting evidence | BP4, BS1, PS3, PM6 |
| *SGCD* | c.212G>C | Uncertain evidence - not enough evidence | PP3 |
| *SYNE1* | c.24422G>A | Uncertain evidence - not enough evidence | BP4, PS3 |
| *TMPO* | c.2068C>T | Likely benign (I) | BS1, PP1, PS3, BP6 |
| *VPS13A* | c.9403C>T | Uncertain evidence - not enough evidence | PP3 |
